# Mother-Newborn Care Unit (MNCU) Experience in India: A Paradigm Shift in Care of Small and Sick Newborns

**DOI:** 10.1007/s12098-022-04145-9

**Published:** 2022-03-04

**Authors:** Harish Chellani, Sugandha Arya, Pratima Mittal, Rajiv Bahl

**Affiliations:** 1 Department of Pediatrics, Vardhman Mahavir Medical College and Safdarjung Hospital, New Delhi, 110029 India; 2 Department of Obstetrics, Vardhman Mahavir Medical College and Safdarjung Hospital, New Delhi, India; 3 Department of Maternal, Newborn & Child, and Adolescent Health, and Ageing, World Health Organization, Geneva, Switzerland

**Keywords:** Mother-newborn care unit (MNCU), Immediate kangaroo mother care (iKMC), Low birth weight (LBW)

## Abstract

While a Cochrane review (2016) showed that kangaroo mother care (KMC) initiated after clinical stabilization reduces mortality by 40%, evidence of the effect of initiating KMC immediately after birth without waiting for babies to become stable was unavailable until recently. This research gap was addressed by a multicountry, randomized, controlled trial co-ordinated by WHO. This trial was conducted in five hospitals in Ghana, India, Malawi, Nigeria, and Tanzania. Implementation of this trial led to development of the “mother–newborn care unit (MNCU).” Mother–newborn care unit or mother–newborn intensive care unit (M–NICU) is a facility where sick and small newborns are cared with their mothers 24 × 7 with all facilities of level II newborn care and provision for postnatal care to mothers. The mother is not a mere visitor, but she has her bed inside the special newborn care unit (SNCU)/newborn intensive care unit (NICU) and as a resident of MNCU, becomes an active caregiver and is involved in continuum of neonatal care. The study results show that intervention babies in MNCU had 25% less mortality at 28 d of life, 35% less incidence of hypothermia, and 18% less suspected sepsis as compared to control babies cared in conventional NICU. World Health Organization is in the process of reviewing the current recommendations on care of preterm or LBW newborns considering new evidence that has become available. However, it would require national policy change to permit mother and surrogate in SNCU/NICU 24 × 7, making the concept of zero-separation a reality.

## Introduction

### Quality Newborn Care in Low-Resource Settings

In developing nations, the quality-of-facility–based newborn care remains a challenge and we need to revisit our strategies for care of small and sick babies to achieve significant reduction in neonatal mortality and morbidity. There are known interventions available which can reduce neonatal mortality, but their coverage is very low.

A notable example is kangaroo mother care (KMC). Most countries have a policy of providing KMC to low-birth-weight (LBW) babies, yet the estimated global coverage of KMC is below 10% [1, 2]. Worldwide, the usual norm is to separate the small sick newborns from their mothers and shift them to neonatal intensive care units (NICU). Mothers are usually discharged or stay in postnatal wards and come to NICU intermittently. The average duration of KMC per day in Indian NICUs is reported to be 3–5 h. This increases to 5–6 h with quality improvement projects [3–5]. Mothers have some access to NICU in most of the centers, but merely as a visitor and not as a caregiver.

### Involvement of Families in Care of Newborns in SNCU/NICU

Studies from developed nations have shown that involvement of parents in care of newborns in NICUs results in better neonatal outcomes [6–8]. There is also evidence to show that lack of involvement of parents in NICU leads to higher stress levels in mothers [9]. On the other hand, if parents stay in NICU for prolonged periods, it leads to improved neonatal outcomes including better rates of breast-feeding, kangaroo mother care, and shorter duration of stay in NICU [8, 10–12]. Active parental involvement also leads to better maternal outcomes in the form of less stress, anxiety, and depression among mothers [12]. A randomized controlled trial from India showed the feasibility of family centered care to complement care of sick newborns [13].

### Kangaroo Mother Care and Low-Birth-Weight Newborns

While a Cochrane review (2016) showed that KMC initiated after clinical stabilization reduces mortality by 40% [6], evidence of the effect of initiating KMC immediately after birth without waiting for babies to become stable was unavailable until recently. This research question was addressed by a multicountry, randomized, controlled trial (iKMC study) co-ordinated by WHO [14]. This trial was conducted in five hospitals in Ghana, India, Malawi, Nigeria, and Tanzania. In India, iKMC study was conducted at Safdarjung Hospital, New Delhi. Infants with a birth weight between 1.0 and 1.799 kg were assigned to receive immediate kangaroo mother care (intervention) or conventional care in an incubator or a radiant warmer until their condition stabilized and KMC thereafter (control). The primary outcomes were death in the neonatal period (the first 28 d of life) and in the first 72 h of life. iKMC stands for immediate and continuous KMC which means KMC to be started soon after birth (within 2 h) and to be given continuously (aiming up to 20 h per day) before and after stabilization. Implementation of this trial led to development of the concept of “mother–newborn care unit (MNCU)”. This article describes setting up of MNCU and its experiences including opportunities and challenges and suggests the way forward.

## Mother–Newborn Care Unit (MNCU)

Mother–newborn care unit (MNCU) or mother–newborn intensive care unit (M–NICU) is a facility where sick and small newborns are cared with their mothers 24 × 7 with all facilities of level II newborn care and provision for postnatal care to mothers. This was necessary in study hospitals to implement iKMC to enrolled babies who all required NICU care because of their birth weight. The mother is not a mere visitor, but she has her bed inside the NICU. Mother as a resident of MNCU becomes an active caregiver and is involved in continuum of neonatal care.

### Setting up of MNCU in India (Both Infrastructure/Design and Services)

**Fig. 1:**
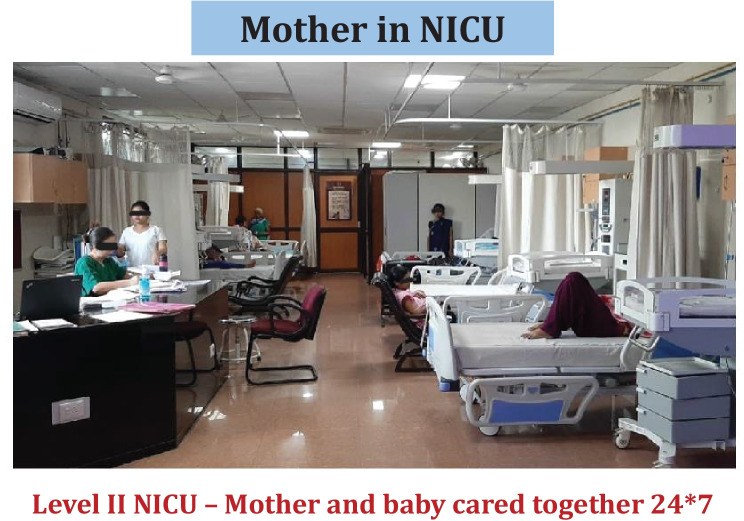
Mother–newborn care unit (MNCU) is level II NICU where mother and baby cared together 24 × 7

**Fig. 2:**
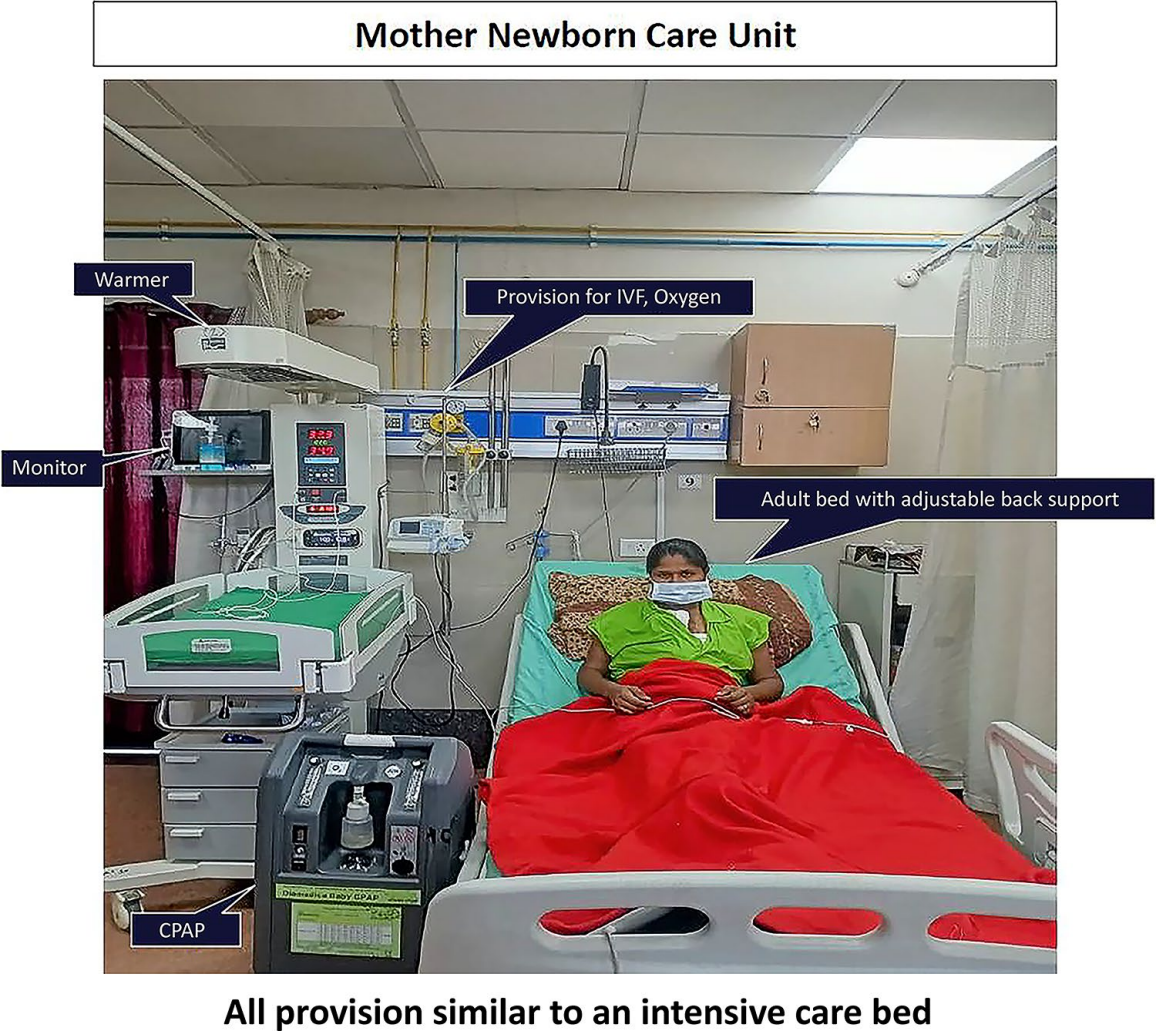
All provisions for level II newborn care

Implementation of immediate KMC required mothers (or surrogates) to be with their babies soon after birth, and continue to be with them 24 × 7 till discharge, i.e., zero-separation. Since there was not enough space in the existing NICU to have mothers’ beds inside, a new NICU was designed with enough space for mother’s bed with each baby [15]. This new NICU was named as "Mother–Newborn Care Unit (MNCU)" (Fig. 1). The infrastructure of MNCU included a toilet, bathing area, pantry, and a clinical examination cubicle for mothers. Like conventional NICU, all equipment for level II intensive care including radiant warmer (required during the time the mother/surrogate could not provide KMC), continuous positive airway pressure (CPAP) machine, oxygen and suction facilities, vital monitor, phototherapy unit, etc. are available (Fig. 2). Mothers were provided postnatal care inside the MNCU and neonatal nurses were trained to provide essential postnatal care to mothers.

### Opportunities for Improved Newborn Care in MNCU

The presence of mother with her baby 24 × 7 in MNCU provided several opportunities to improve newborn care.

#### Prolonged, Continuous, Effective KMC

In the iKMC study, the median daily duration of skin-to-skin contact in the neonatal intensive care unit was 16.9 h (interquartile range, 13.0 to 19.7) in the MNCU and 1.5 h (interquartile range, 0.3 to 3.3) in the conventional NICU [14].

#### Higher Breast-Milk Feeding Rates

A very important opportunity that MNCU provides is early exclusive breast-milk feeding and breast-feeding. Since mother is with her baby in MNCU, expressed breast-milk (EBM) is readily available as first feed for initiation soon after birth. Skin-to-skin contact with baby results in better lactation and it is easier to maintain babies on exclusive breast-milk feeding. Babies can be put to the breast earlier for non-nutritive sucking (NNS), which helps babies to develop reflexes faster and improves milk output of the mother by stimulating prolactin reflex. In the iKMC study, breast-milk feeding was initiated within the first 24 h after birth in 58.5% of infants in the MNCU and in 45.5% of infants in the conventional NICU; full breast-feeding occurred within 7 d in 78.4% infants in MNCU vs. 69.0% of infants in conventional NICU [14].

#### Mother as Caregiver and Not a Mere Visitor

In resource-limited countries like India, due to low nurse–baby ratio, it is difficult to provide quality care to neonates. Mothers in MNCU substantially contribute to care of babies including feeding, changing diapers, and monitoring babies for danger signs. MNCU provides opportunity for mother to be the primary caregiver in MNCU, thus providing family-centered developmentally supportive care to newborns. Presence of mother in MNCU gives ample opportunity to health care personnel to teach the mothers, healthy practices of neonatal care thus preparing them for taking care of neonates after discharge. Mothers in MNCU have less anxiety and stress as compared to mothers staying away from their babies in postnatal ward. Last but not the least, MNCU resulted in mother–newborn couplet care by pediatrician and obstetrician with better co-ordination of neonatal and maternal care.

### Outcomes in MNCU

Neonatal deaths in the first 28 d of life occurred in 191 of 1596 infants in the intervention group (12.0%) and in 249 of 1587 infants in the control group (15.7%) [relative risk of death, 0.75; 95% confidence interval (CI), 0.64 to 0.89; *p* = 0.001], i.e. 25% less risk of mortality in MNCU as compared to NICU [14]. This implies that 1,50,000 neonatal deaths can be prevented globally if this model of care is adopted.

The study results also show that intervention babies in MNCU had 35% less incidence of hypothermia and 18% less suspected sepsis as compared to control babies cared in conventional NICU. There are several possible reasons for the lower risk of infections, including increased breast-milk feeding, lower risk of cross infection since one mother cares for only her baby rather than a nurse caring for 8–12 babies, and colonization of the baby with bacteria from the mother’s microbiome rather than the NICU environment.

### MNCU Challenges

There were several challenges, which we faced and overcame in providing care in MNCU.

#### Initiating KMC in Delivery Area, Transporting to MNCU

iKMC stands for immediate and continuous KMC after birth. Mothers need to be observed for about 2 h after vaginal delivery and 6 h after cesarean section, and most enrolled 1.0 to 1.8 kg babies need early transfer to NICU for monitoring and management. This challenge was overcome by having a surrogate (family member) chosen by the mother available in the delivery area for transporting baby to MNCU in KMC position. In the MNCU, the surrogate provided KMC till mother reached MNCU. Having a family member next to mother in the labor room additionally contributed to support and respectful care for the mother.

#### Respiratory Support Provision in KMC Position

**Fig. 3:**
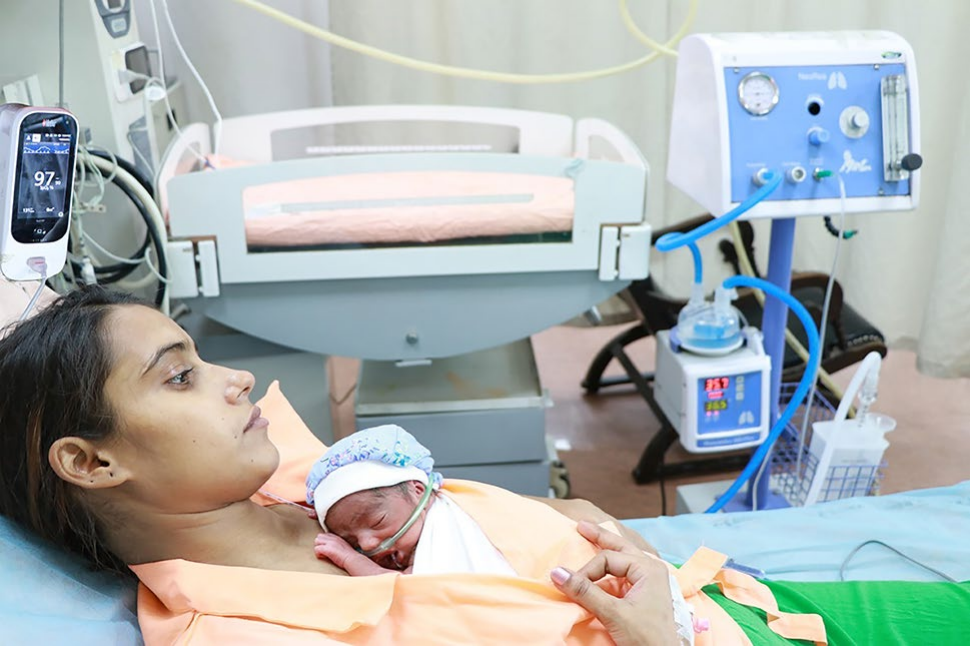
Providing continuous positive airway pressure (CPAP) in KMC position

Majority of babies < 1.8 kg are preterm and many of them develop early respiratory distress requiring respiratory support in the form of CPAP. Learning to provide CPAP in KMC position was an important challenge [16]. Nasal Interface for CPAP was optimized, and standard operating procedures for fixing it appropriately were developed and implemented (Fig. 3). A binder was used to maintain the baby’s neck in a slightly extended position. A pulse oximeter was constantly used when the baby was in KMC position to monitor heart rate and oxygen saturation, so that any sudden changes in vitals could be detected.

#### Care for the Mother in MNCU

Providing care to mothers from a few hours after birth was an important challenge in MNCU. An essential maternal–postnatal care package was developed and neonatal nurses were trained in implementing this package. Obstetricians took daily rounds for mothers and attended immediately to their urgent needs. We learnt from experience of mother–newborn couplet care in Sweden [17, 18]. A strong co-operation, co-ordination, and collaboration between pediatricians and obstetricians is the cornerstone of MNCU.

#### Providing Continuous KMC (Aiming for at Least 20 Hours a Day)

There are several challenges providing continuous KMC (aiming for at least 20 h a day) in MNCU. Most common reason for separation was mother being not available due to medical reasons or for daily routines like bathing, toilet, etc. This challenge was overcome with the help of surrogate who provided KMC in MNCU when mother was not available. Another common reason for separation during iKMC is medical procedures and treatment of baby including phototherapy. Some procedures like glucose monitoring, gavage feeding, giving IV injections can be done even while the baby being in KMC position. However, other procedures like inserting IV cannula, fixing CPAP cannula, putting orogastric tube, phototherapy, etc. required separation, but the baby was immediately placed in KMC position following the procedure.

#### Experience from Other Countries

Besides India, other countries which participated in iKMC study were Ghana, Nigeria, Malawi, and Tanzania [14]. Most of these sites had experience similar to that of India. Ghana, Malawi, and Nigeria changed half of their NICUs to MNCUs while Tanzania (like India) built a separate MNCU. Finding surrogates was challenging in many of the sites. Similarly, transferring mothers early to MNCUs (particularly after cesarean section) was a challenge in most of these sites.

### Sustenance of MNCU after iKMC Study

After completion of the study, MNCU facility has been continuing at Safdarjung Hospital, New Delhi, based on the positive experience of care providers and families, as well as evidence of substantial improvement in outcomes. MNCU at Safdarjung Hospital is a 12-bedded level II NICU, where babies weighing less than 1800 g are admitted and their mothers stay with the babies 24 × 7 in MNCU.

#### Experience During COVID Pandemic

**Fig. 4:**
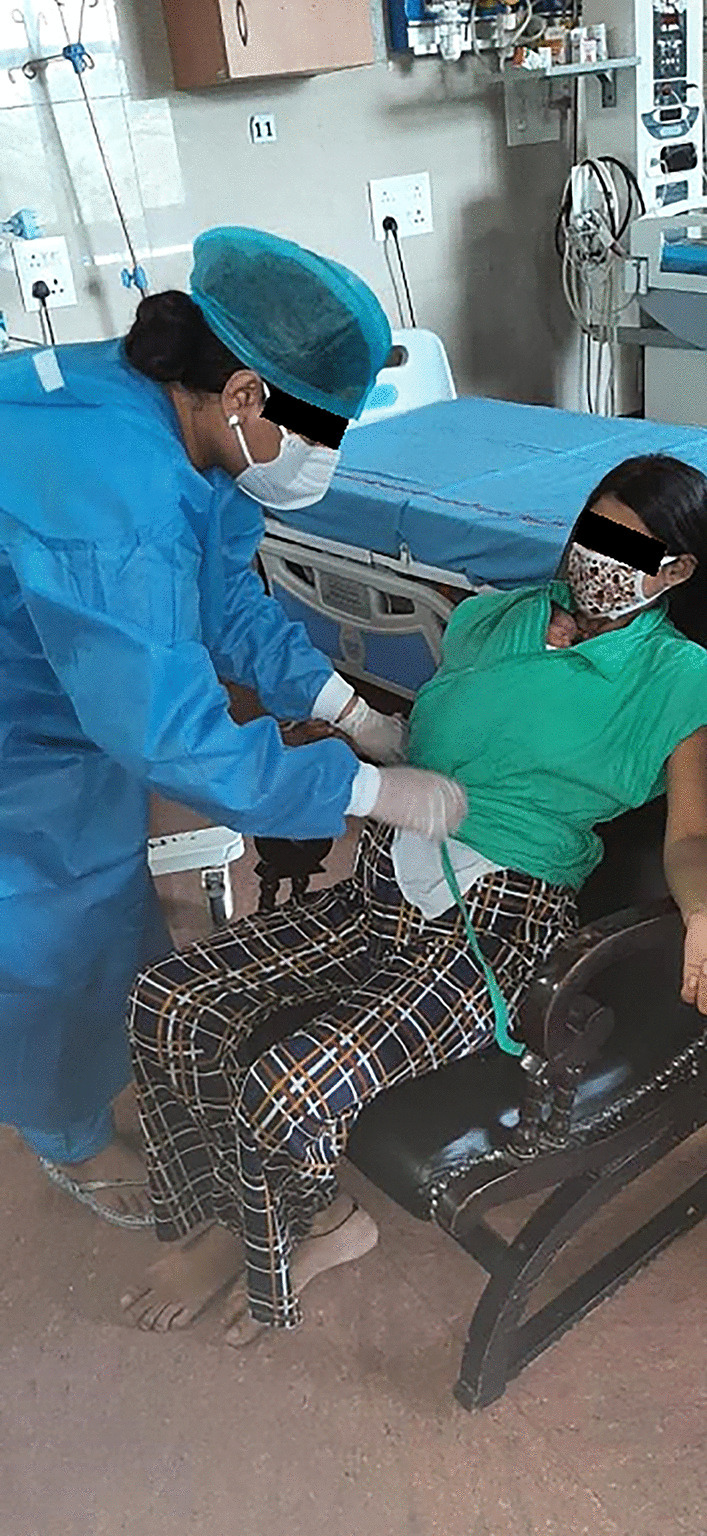
Mother–newborn care unit (MNCU) during the COVID pandemic

In March 2020, when COVID-19 cases started increasing in India, there were apprehensions among health professionals as well as patients regarding spread of infection in MNCU. To overcome this challenge following measures have been taken. First, all mothers are screened at the time of delivery and only COVID-negative mothers are transferred to MNCU. Second, all COVID-appropriate behaviors are ensured in MNCU, including strict use of mask, hand hygiene and respiratory hygiene (Fig. 4). If any mother develops symptoms suggestive of COVID infection, she is shifted immediately to suspected COVID area, investigated and managed accordingly. With these measures, we have been running this facility successfully throughout the ongoing pandemic with 100% occupancy of 12 mothers with 12 to 18 babies, as many of these mothers have twin babies.

## Way Forward

Health care providers have been separating small and/or sick babies from their mothers for decades believing that is best for them. New evidence summarized in this paper suggests we must establish the practice of zero separation of mothers and newborns globally. Presence of mother in NICU 24 × 7 is a paradigm shift in the care of small and sick babies.

This will need infrastructure changes. The main issue in most of the hospitals is nonavailability of adequate space. Mother’s bed which is presently in postnatal ward needs to be placed inside NICU which needs reorganization of infrastructure. New special newborn care units (SNCUs) in district hospitals and NICUs in tertiary care hospitals should be designed with all the provisions for mother to stay 24 × 7 as a caregiver to make them MNCU. Similarly, we should adopt this new design when renovating already functional NICUs and SNCUs.

This will also need certain policy changes, i.e., allowing mothers/surrogates in MNCU (same as that for family-centered care), shifting small babies from delivery areas to MNCU in KMC position, obstetric rounds inside MNCU, and giving essential care to mothers in MNCU by neonatal nurses. Pediatricians, obstetricians and policymakers need to be taken into confidence and convinced of the benefits and feasibility of MNCUs for this paradigm shift in care of small and sick newborns. It also involves strong collaboration between Pediatrics and Obstetrics department.

World Health Organization is in the process of reviewing the current recommendations on care of preterm or LBW newborns considering new evidence that has become available [19]. However, it would require national policy change to permit mother and surrogate in NICU 24 × 7, making the concept of zero-separation a reality.
